# Impact of mutations on the stability of SARS-CoV-2 nucleocapsid protein structure

**DOI:** 10.1038/s41598-024-55157-8

**Published:** 2024-03-11

**Authors:** Nelli Muradyan, Vahram Arakelov, Arsen Sargsyan, Adrine Paronyan, Grigor Arakelov, Karen Nazaryan

**Affiliations:** 1 Laboratory of Computational Modeling of Biological Processes, Institute of Molecular Biology of the National Academy of Sciences of the Republic of Armenia (NAS RA), 0014 Yerevan, Armenia; 2 Russian-Armenian University, 0051 Yerevan, Armenia

**Keywords:** Computational models, Molecular modelling, Protein structure predictions

## Abstract

The nucleocapsid (N) protein of SARS-CoV-2 is known to participate in various host cellular processes, including interferon inhibition, RNA interference, apoptosis, and regulation of virus life cycles. Additionally, it has potential as a diagnostic antigen and/or immunogen. Our research focuses on examining structural changes caused by mutations in the N protein. We have modeled the complete tertiary structure of native and mutated forms of the N protein using Alphafold2. Notably, the N protein contains 3 disordered regions. The focus was on investigating the impact of mutations on the stability of the protein's dimeric structure based on binding free energy calculations (MM-PB/GB-SA) and RMSD fluctuations after MD simulations. The results demonstrated that 28 mutations out of 37 selected mutations analyzed, compared with wild-type N protein, resulted in a stable dimeric structure, while 9 mutations led to destabilization. Our results are important to understand the tertiary structure of the N protein dimer of SARS-CoV-2 and the effect of mutations on it, their behavior in the host cell, as well as for the research of other viruses belonging to the same genus additionally, to anticipate potential strategies for addressing this viral illness․

## Introduction

The SARS-CoV-2 virus contains a single-stranded RNA genome packaged in a 100-nm-diameter membrane-bound virion. The viral genome is made up of a positive sense, single-stranded RNA that encodes four structural proteins—spike (S), envelope (E), membrane (M), and nucleocapsid (N)—along with 9 auxiliary proteins and 14 open reading frames (ORFs) that encode 16 nonstructural proteins, making up a replicase complex. One of the most abundant structural proteins in cells infected with viruses is protein N, which is very conservative in the CoV genus^1–3^.

The primary function of the N protein is to encapsulate the viral RNA into long-stranded ribonucleocapsid (RNP) complexes, as well as to participate in the assembly of the virus by interacting with the M protein of the virus genome. Additionally, it has been demonstrated that the CoV N protein participates in host cellular processes that control the viral life cycles, also the N protein is an immunodominant antigen of the host cell immune system^1,3–5^.

The N protein is encoded by the 9th ORF of the virus, consists of 419 amino acids, and has a modular structure that can be divided into intrinsically disordered regions (IDRs) and conservative structural regions^2,4^. IDRs include 3 modules: N-arm (1–48 aa), central Ser/Arg rich linker region (LKR) (176–247aa), and C-tail (366–419 aa), while conservative structural regions include two modules: N-terminal domain (NTD) (49–175 aa) and C-terminal domain (CTD) (248–365 aa). In the primary structure, NTD and CTD are connected by LKR and are surrounded by N-arm and C-tail^1,3^.

The presence of disordered regions in viral proteins is commonly linked to viral infectivity and pathogenicity. Disordered regions participate in liquid–liquid phase separation, which is also typical of N protein, which exhibits concentration-dependent liquid–liquid phase separation in the presence of RNA^6,7^.

The N protein is known to exhibit functional activity when present in a dimeric structure^1,8^. In the host cell, the N protein typically undergoes phosphorylation, mainly occurring in its linker region. Additionally, it interacts with several viral proteins such as Membrane (M ) and non-structural protein (Nsp33), and host proteins including GTPase-activating protein SH3 domain-binding protein 1/2 (G3BP-1/2), heterogeneous nuclear ribonucleoproteins (hnRNPs), NLR family PYRIN domain containing-3 (NLRP3), 14-3-3, and others.^3,6,9^.

The data revealed that the regions spanning amino acid positions 164 to 205, exhibited the highest number of mutations compared to the total number of amino acids among the N amino acid sequences, and the second-highest frequency of mutations was observed in the regions spanning amino acid positions 205 to 246^10^. Some mutations can have a positive impact on pathogenesis, such as R203K/G204R mutations^11–13^, but at the same time, these mutations decrease the structural stability and flexibility of the N protein. Also, there are data about the absence of a significant correlation between N protein mutations and infection rates^14^. Some variants containing the R203K/G204R mutations in the N protein often exhibit concurrent mutations, including the spike protein’s N501Y and E484K, among others^13^.

Based on the above-mentioned, it is indeed challenging to establish a clear correlation between specific mutations and pathogenesis or disease severity. Taking into account that viral proteins do not mutate singularly, and the impact on pathogenesis is likely influenced by the combination of multiple mutations in different proteins, it becomes a complicated task. Therefore, determining the relationship between mutations and disease outcomes requires a comprehensive analysis of various mutation combinations rather than focusing on individual mutations in isolation^12,15^.

Due to the presence of multiple disordered regions within the N protein, including the N-arm, LKR, and C-tail^1,2,4^, acquiring its comprehensive tertiary structure through experimental techniques like X-ray crystallography has presented formidable challenges^16–18^. As of now, there is no established protocol for elucidating the structural conformation of disordered proteins. However, it's pertinent to highlight that the complete monomeric structure of the N protein became accessible in the Protein Data Bank (PDB) on January 2023, utilizing electron microscopy with a resolution of 4.57 Å (PDBID: 8FD5).

Herein, we used computational approaches to understand how mutations impact the stabilization of SARS-CoV-2 N protein dimeric structure. We performed molecular modeling for the complete tertiary structure of wild-type and mutated N proteins, molecular dynamic simulations, binding free energy and RMSD (root mean square deviation) calculations, and structural rearrangements among wild-type and mutated proteins. The data obtained from this study are essential to understanding the tertiary structure of the N protein of SARS-CoV-2 and the impact of mutations on protein dimeric structure. Moreover, the findings shed light on the behavioral dynamics of these mutations within host cellular environments. Such insights are crucial and could expand the scope of research on viruses of a similar genus.

## Results

We used Alphafold2 (AF) for modeling and as we mentioned, there is no X-ray crystallographic model of the N protein complete tertiary structure, only 2 domains are available, thus we based our further validation process on 2 known X-ray crystallographic domains: NTD (*PDBID:7N0R*), and CTD (*PDBID:6WZQ*).

**Figure 1:**
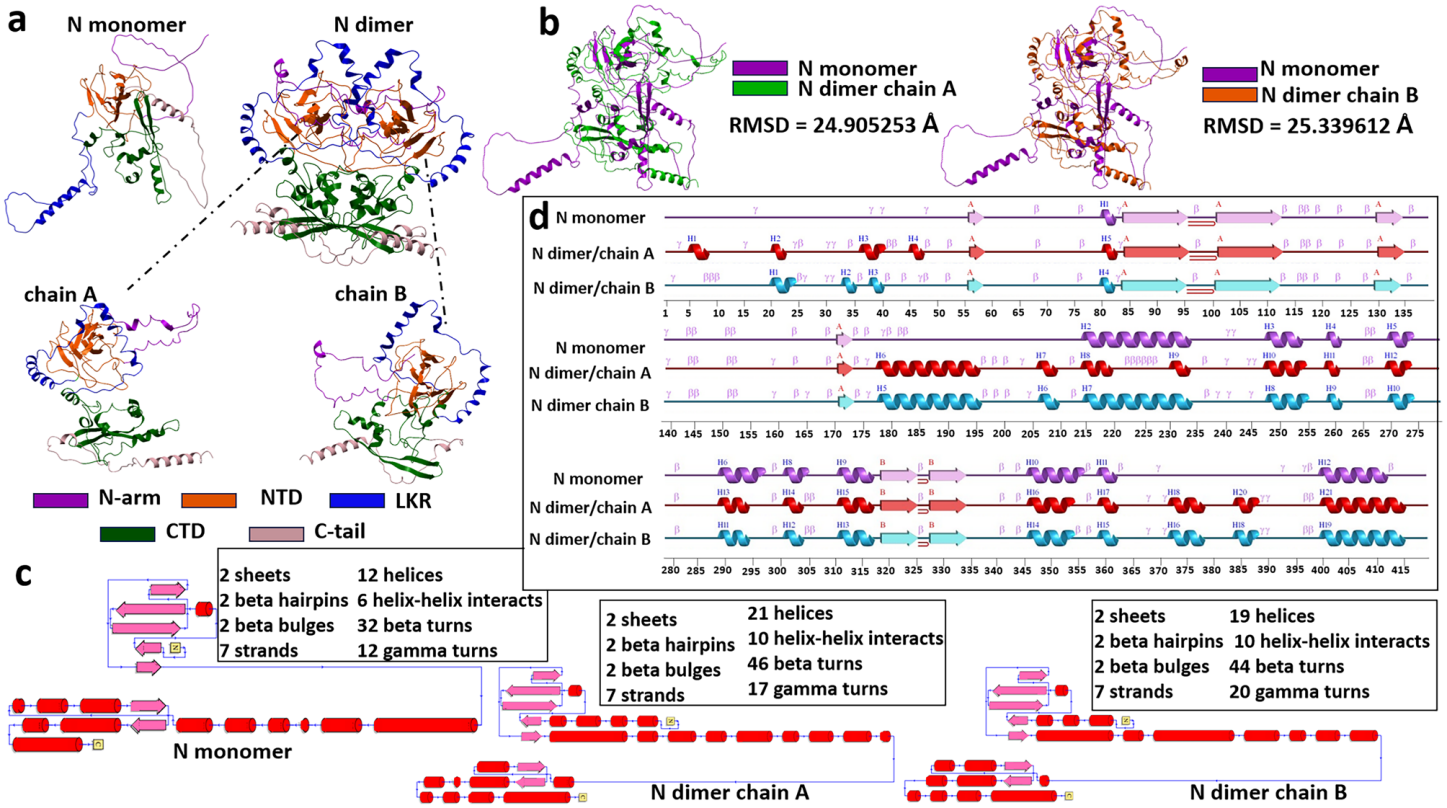
Modeled N protein structure. (**a**) N monomer structure, N dimer structure with monomer 1 (chain A) and monomer 2 (chain B). (**b**) superimposed structures of N monomer with N dimer chain A and N dimer chain B, (**c**) topology structures with descriptions, d- secondary structure elements of N monomer, N dimer chain A and N dimer chain B, (**a**,**b**) generated by Molsoft L.L.C.: ICM-Pro version 3.9-2c, (**c**) and (**d**) by PDBsum Generate (ebi.ac.uk).

Figure 1a represents the N protein monomer, dimer, dimer’s chain A and chain B complete modeled structures. It can be seen from the tertiary structure that the monomer disordered regions (N-arm, LKR, C-tail), besides not having the characteristic elements of the secondary structure are scattered and not compactly assembled, and in the case of the dimer, the structure is different. We separated the dimer into 2 monomers (chain A and chain B) that make up the dimer and compared them with the N monomer (Fig. 1b). Superimposition of modeled proteins on these domains and RMSD score comparison was performed using ICM Pro software. RMSD comparison showed that RMSD [AF_N_monomer vs AF_N_ dimer_chainA] = 24.905253 Å and RMSD [AF_N_monomer vs AF_N _dimer_chainB] = 25.339612 Å.

**Figure 2:**
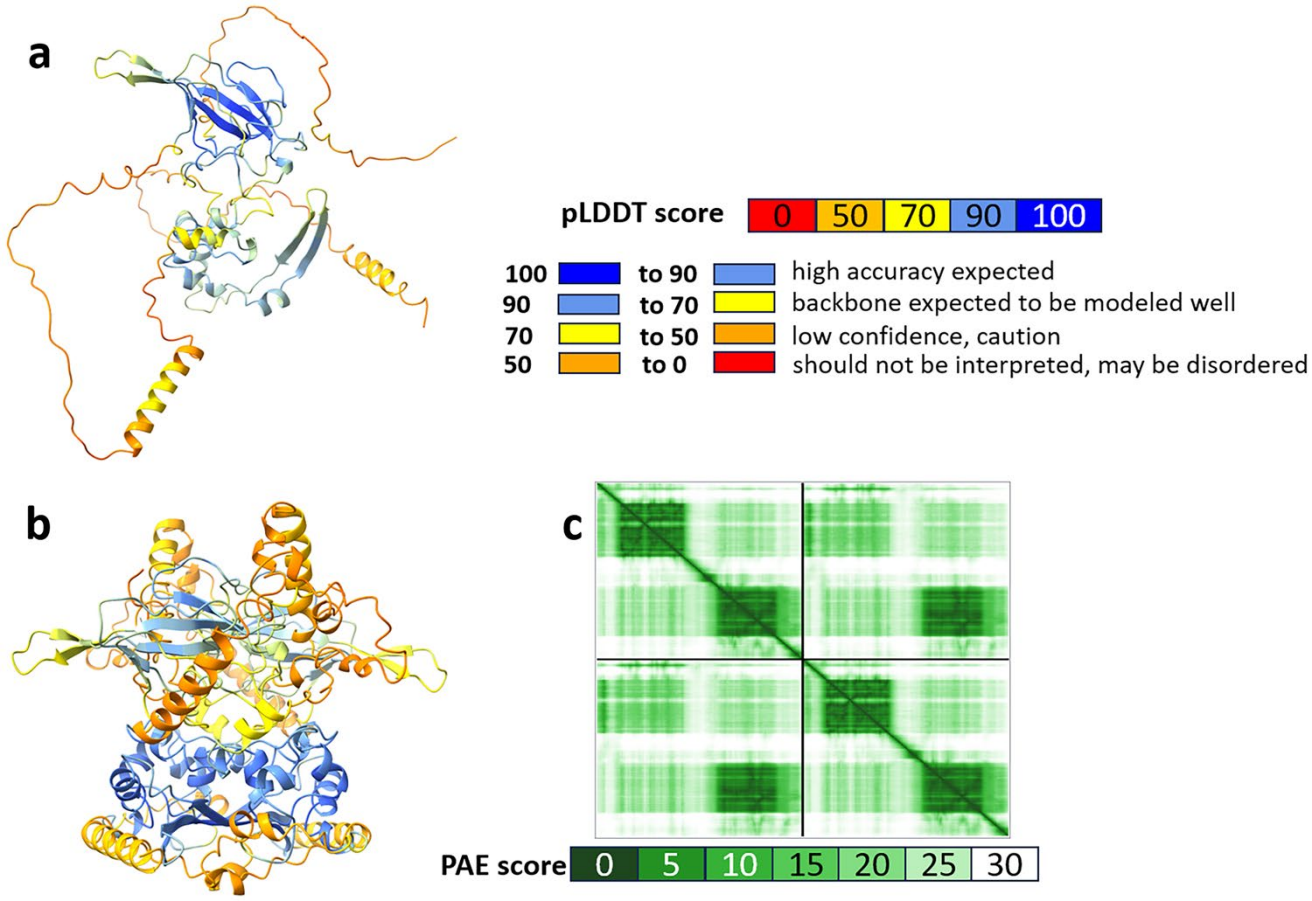
Evaluating the accuracy of model predictions. pLDDT confidence measure in the B factor field (**a**) N monomer, (**b**) N dimer, (**c**) PAE plot for N dimer, images were generated by UCSF ChimeraX, version 1.6.1.

Given the limitation of observing structural rearrangements within tertiary structures, we opted to visualize these alterations in secondary structural elements as a more effective means of analysis and visualization. We used the PDBSum server to understand what kind of elements of secondary structures are presented in the modeled protein. The differences in the structure are also shown by the analysis of the topology and the secondary structure elements (Fig. 1c,d). It is important to note that in both monomer and dimer models, there is an alpha helix between 400 and 412 amino acids (Fig. 1d), which is the C-tail and one of the disordered regions of N protein. In the case of dimer, there are several short alpha helixes formed, the most noticeable of which are between 178 and 196 amino acids in chain A and B and between 215 and 234 amino acids in chain B and monomer (Fig. 1d). Figure 2a and b represent the pLDDT confidence measure in the B factor field obtained by ChimeraX. The pLDDT score represents a pivotal instrument within the domain of protein structural prediction. Its primary function is to provide an estimate of the confidence levels associated with individual amino acid residues within predicted protein configurations․ Employing a continuum ranging from 0 to 100, with scores exceeding 90 signifying a robust degree of assurance and those falling below 50 indicating a diminished level of confidence, the pLDDT score plays a critical role in visualizing AlphaFold2 models. Specifically, it imparts a color scheme where regions of heightened confidence are typically rendered in gradients of blue, contrasting with lower confidence regions, which are often portrayed in the hues of yellow, orange, or red. The pLDDT score evaluates whether the predicted positioning of a given residue aligns with the distances between its C-alpha atom and neighboring C-alpha atoms (within a 15 Å range) as observed in the true protein structure^19^. In the computational models presented (Fig. 2a and b), regions characterized by hues of yellow and orange signify pLDDT scores ranging from 50 to 70 which indicate IDRs within the protein structure. The manifestation of such IDRs is a documented attribute and is deemed characteristic within this molecular framework. The N monomer exhibited an average pLDDT score of 65.54, while the dimer was characterized by an average iptm + ptm score of 0.466. The PAE plot functions similarly to a map, highlighting the differences between the predicted and actual positions of various segments of a protein. PAE represents the absolute error in the relative positioning of residues, quantified in Ångströms (0–30)․ Colors are used to represent these disparities, with dark green indicating minimal differences and white representing larger discrepancies. When examining this map, we expect most points along the diagonal line to be close to dark green because this is where the predicted and actual positions should closely align. If we observe distinct clusters of similar colors elsewhere on the map, it signifies that the model is highly confident in those regions. An outstanding model would display an entirely dark green map, signifying highly accurate predictions of the protein's structure^19^. The plot c displayed in Fig. 2c represents a PAE plot specifically designed to depict the characteristics of the N-dimer model.

**Figure 3:**
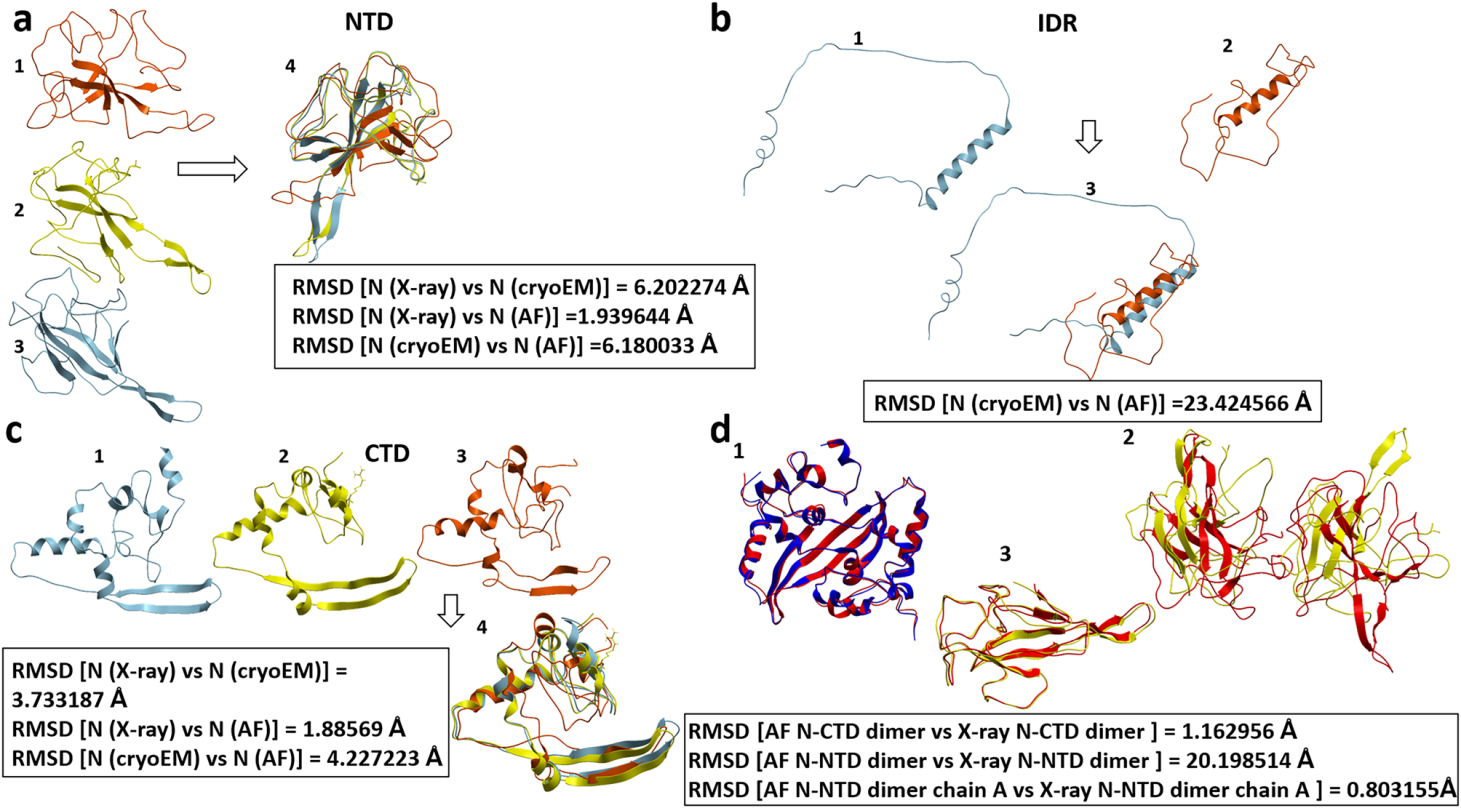
N protein structures comparison. (**a**) NTD- 1- AF monomer model, 2-X-ray model (PDBID:7N0R), 3- CryoEM model (PDBID:8FD5), 4-superimposition of NTD domains for AF, X-ray and CryoEM models. (**b**) IDR/LKR—1-AF model, 2-CryoEM model, 3-the superimposition of IDRs for AF and CryoEM models, (**c**) CTD—1-AF monomer model, 2-X-ray model (PDBID:6WZQ), 3- CryoEM model, 4-superimposition of CTD domains for AF, X-ray and CryoEM models, (**d**) 1-CTD dimer (Red-AF, blue-X-Ray), 2-NTD dimer (red-AF, yellow-X-ray), 3-NTD monomer (red-AF, yellow-X-ray), images were generated by Molsoft L.L.C.: ICM-Pro, version 3.9-2c.

On January 2023 Casasanta M et al. released the N protein monomer model using cryo-electron microscopy (Cryo-EM) (*PDBID:8FD5*) with 4.57 Å resolution^20^. Our investigation entailed a comprehensive examination of the N monomers' domains within our AF models in superimposition with the analogous domains derived from Cryo-EM and X-ray crystallography methodologies. The results are presented in Fig. 3.

To check the accuracy of the AF models we compared them with X-ray and with CryoEM structures (Fig. 3a–c). Through calculations and comparisons, we have established the significant utility of AF modeling. Specifically, when we superimposed AF-NTD and AF-CTD models onto X-ray domains, we observed remarkably low RMSD scores (Fig. 3a and c), measuring at 1.94 Å and 1.89 Å, respectively. However, in the case of IDR, where no X-ray model was available for reference, we conducted a comparison solely with the Cryo-EM model (Fig. 3b), resulting in a considerably higher RMSD score exceeding 23.4 Å. This elevated RMSD score can be attributed to the absence of structured regions in the AF and Cryo-EM models, with Cryo-EM models demonstrating greater assembly of disordered regions, a behavior consistent with expectations.

The N protein acquires functional activity when it adopts a dimeric structure. Consequently, in subsequent experiments, we used the AF dimeric configuration. To assess the reliability of the AF dimeric structure, we considered not only the pLDDT and PAE scores but also examined whether its dimerization domain contributed to the formation of the dimer. Furthermore, we conducted a comparison of structures with the X-ray model (PDBID:6WZQ, PDBID:7N0R), which was originally associated with the oligomeric structure (Fig. 3d). When comparing the AF-NTD dimeric structure with X-ray dimeric structures, we observed a higher RMSD score for the NTD dimer as represented in Fig. 3d. This elevated RMSD score can be attributed to the fact that the NTD does not participate in dimerization. However, in a separate comparison where we compared the NTD-dimer chains with X-ray monomeric structures, we found a significantly lower RMSD score of 1.16 Å. This lower RMSD score suggests a closer structural resemblance between the NTD-dimer chains and the X-ray monomeric structures. In contrast, for the CTD dimer, the RMSD score was 0.8 Å, which aligns with our expectations. This low RMSD score indicates a close match between the AF-CTD dimeric structure and the X-ray dimeric structures, suggesting that the CTD is actively involved in dimerization.

We evaluated several metrics such as pLDDT, PAE, RMSD scores, along with tertiary and secondary structure elements, and topologies. These metrics uniformly confirmed the precision and dependability of the AF models. Apart from the precision concerns of AF modeling, our main objective was to understand how mutations in the N protein impact the stability of its dimeric formation.

For our study, we chose 34 single mutations (D3L, Q9L, P13L, D63G, I157T, Q160R, P168Q, A173S, R185C, S186F, S197A, S197L, S202N, R203E, R203M, T205A, T205I, A208S, G215C, S235F, K256N, T265I, A267V, T296I, F307V, A308S, M322I, P326L, K374N, D377Y, Q384H, D401Y, S413I, Q418H) and 3 combined mutations, each distinctive to specific strains of concern. These include the Alpha strain (lineage B.1.1.7) with mutations D3L, S235F, R203K, G204R; the Gamma strain (lineage P.1) and Omicron strain (lineage B1.1.529) both featuring R203K, G204R mutations, and the Delta strain (lineage B1.617.2) identified by mutations D63G, R203M, D377Y.

**Figure 4:**
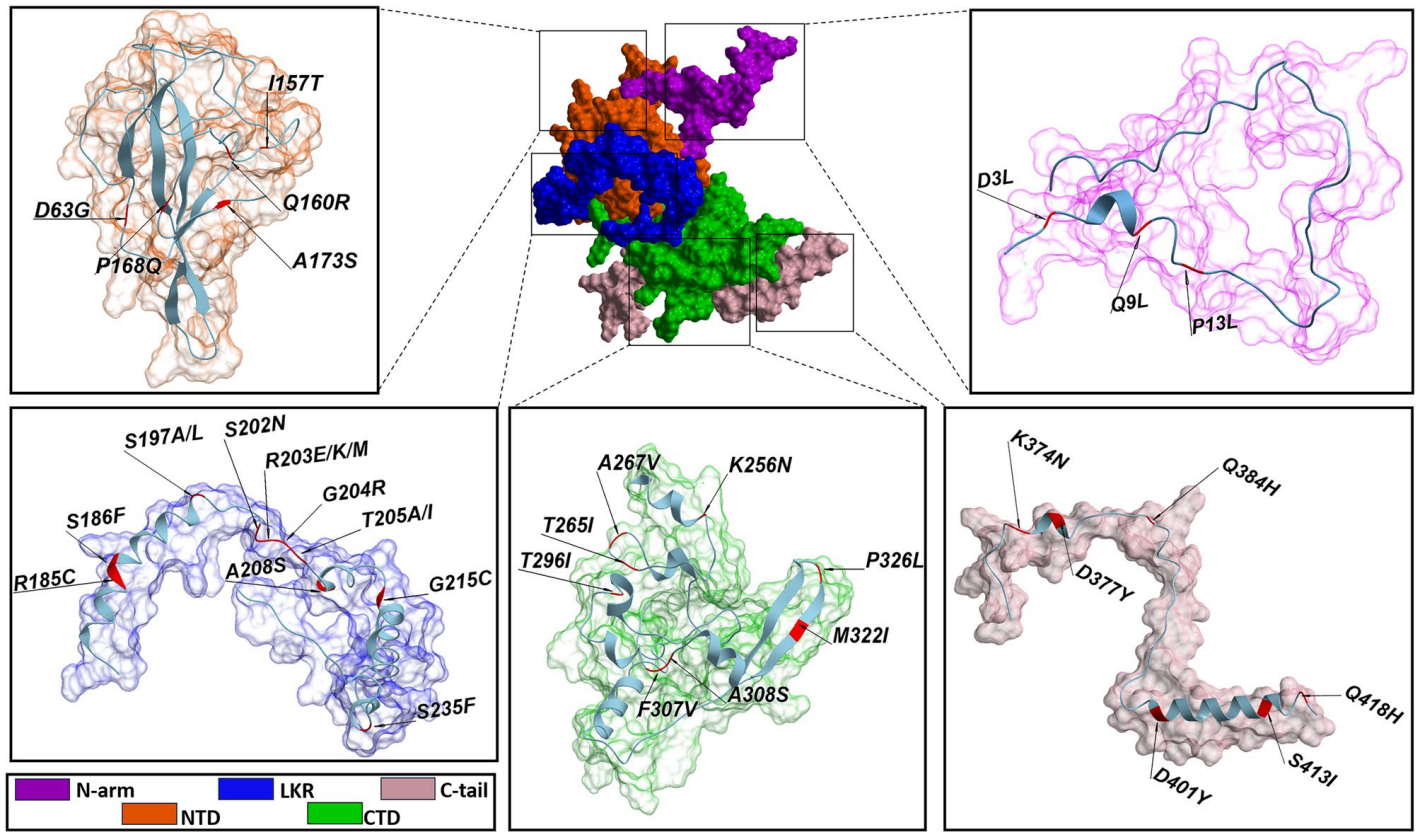
N protein mutations by their localization. Represented tertiary structures are for N wild type, images were generated by Molsoft L.L.C.: ICM-Pro, version 3.9-2c.

It is also important to note that the R203K/G204R mutants occur together^10,16^, and we also observed this in the single-protein model. T205I is associated with the Beta strain (lineage B.1.351) so we observed T205I single mutation as a Beta strain-related mutation. Thus, we have had 38 models, 1 for native N protein (N wild type) and 37 for mutated proteins. Data for N protein mutations were obtained from the previous study^21^ and the CoV-Lineages Report^22^. Figure 4 represents all selected mutations for further study- according to their location: it can be seen that the vast majority of mutations are located in the disordered regions N-arm -3 mutations, LKR-14 mutations, C-tail-6 mutations, and in the functional domains—NTD-5 and CTD-8 mutations. For further experiments, the native N protein was chosen as the control model. The supplementary Table S1 represents the RMSD scores for all models compared with the native N protein structure.

**Figure 5:**
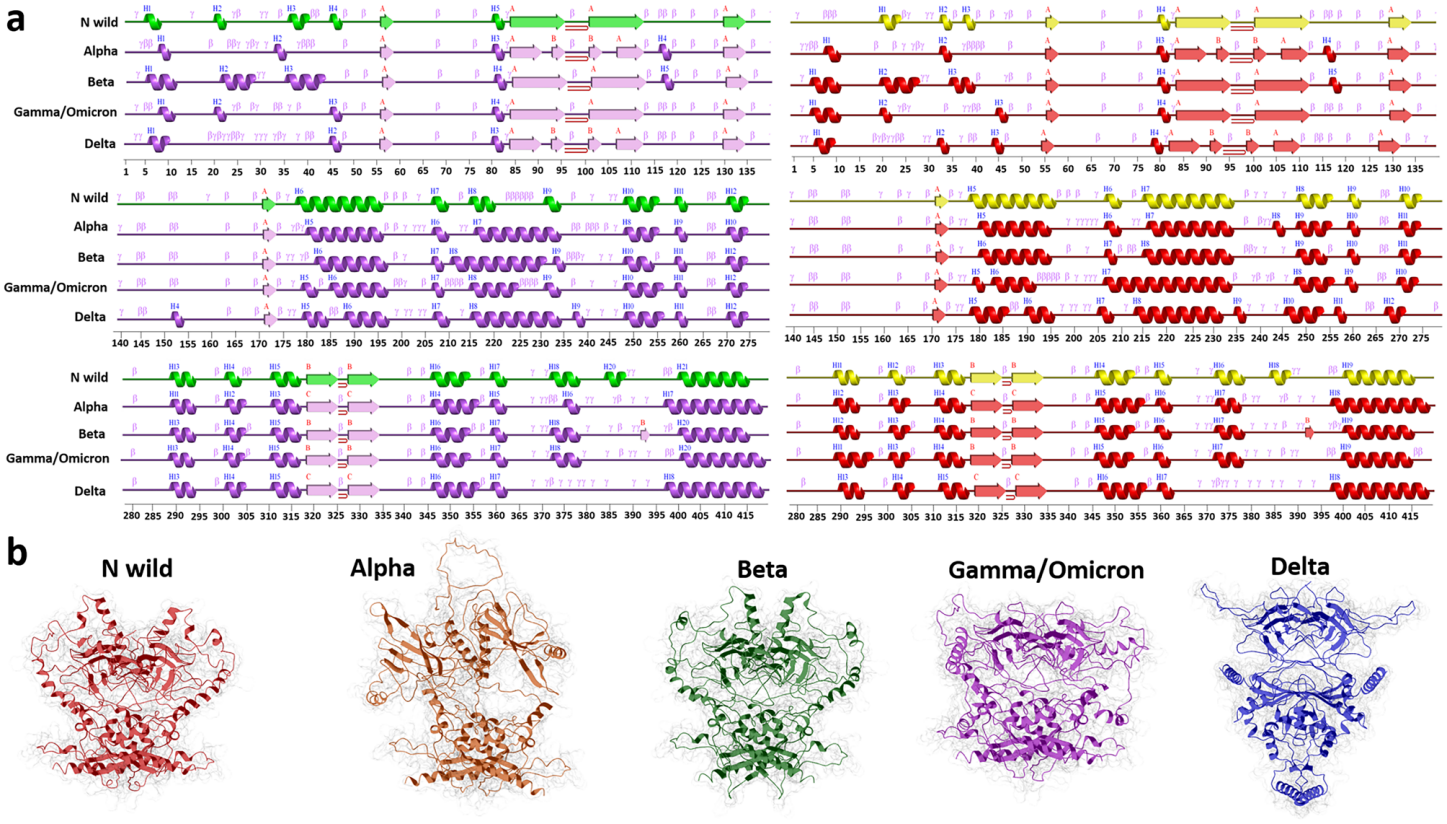
Strain-specific protein tertiary structures and secondary structure elements. (**a**) Secondary structure elements, each dimer was separated into 2 monomers (green and yellow for N wild type, violet and red for mutated models), (**b**) tertiary structures for strain-specific models, image (**a**) was generated by PDBsum Generate (ebi.ac.uk), image (**b**) generated by Molsoft L.L.C.: ICM-Pro version 3.9-2c.

The strain-specific protein tertiary structures were looked at, as shown in Fig. 5. The data analysis reveals notable distinctions between the mutant models and the native N protein structure, as observed through structural superimposition. Figure 5a represents the existence of variations in the secondary structure elements within each strain-specific model which illustrates the impact of the mutation on the structure. All mentioned rearrangements affect on tertiary structure of strain-specific models (Fig. 5b).

An in-depth examination of the mutated variants revealed that all the studied mutations had an impact on the secondary structure elements of the protein, resulting in structural rearrangements such as loop-to-α-helix, loop-to-β-sheet, β-sheet-to-loop, and α-helix-to-loop transitions, as shown in Supplementary Fig. S1. We employed the PDBSum website for this structural analysis^23^. All studies were performed at the level of tertiary structure, but for visualization and analysis, the tertiary structure was represented as a secondary structure. Since the server performs calculations at the monomer level, we separated the model dimers by monomer chains and then considered them.

The models utilized in our research were selectively phosphorylated at several distinct sites: S23, S180, S186, S188, S194, S197, S201, S202, S206, T24, T198, T205, T265, and T391. After the phosphorylation process, these modified structures were then subjected to MD simulations. Each simulation was conducted for a substantial duration of 100 ns with a simulation time step of 2 fs.

**Figure 6:**
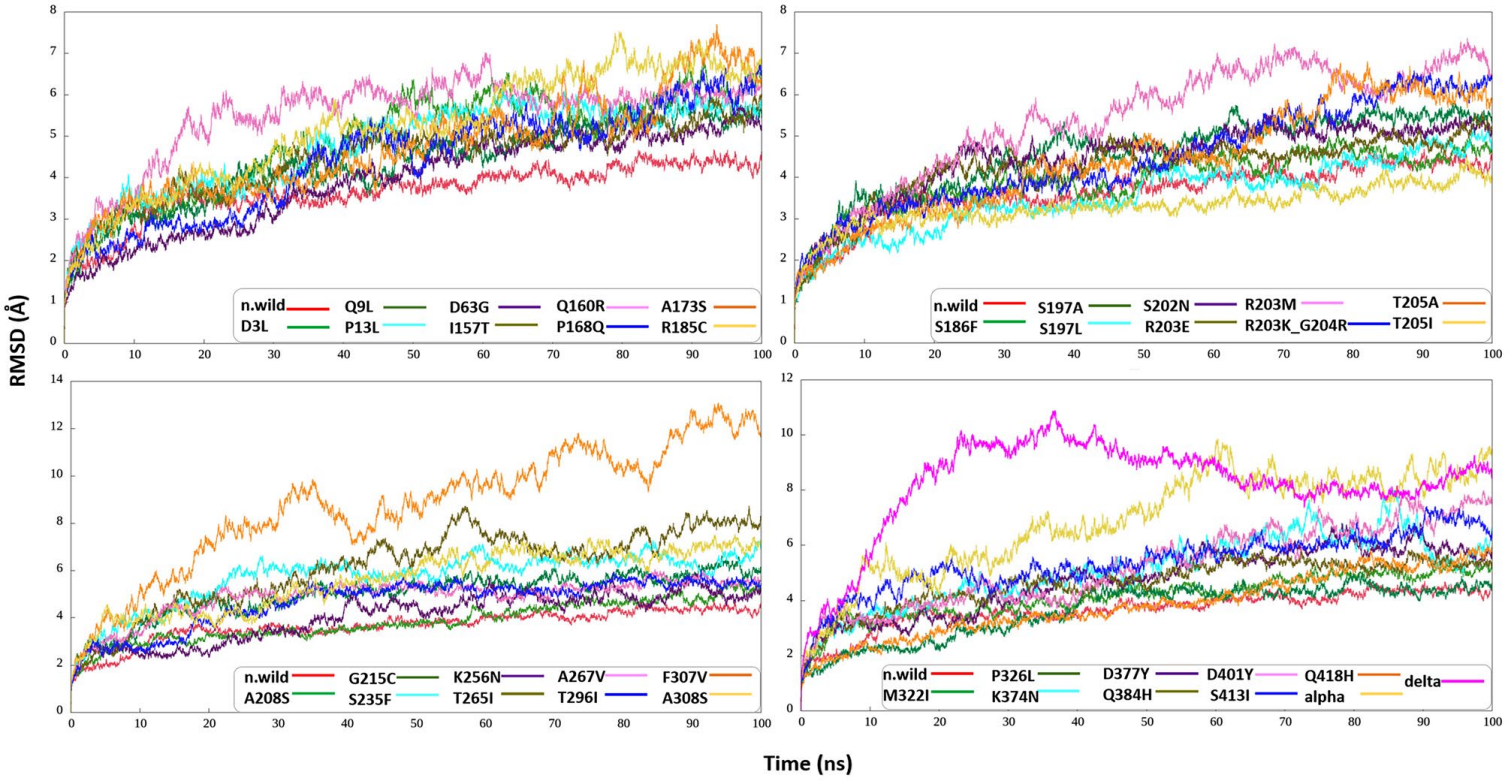
RMSD as a function of time. In each graph, red line represents RMSD fluctuation for N-wild type.

**Figure 7:**
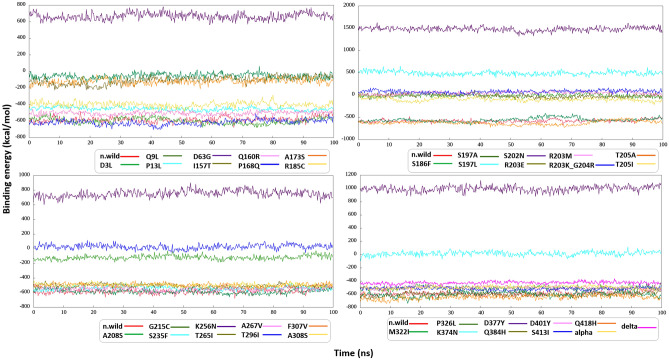
Binding free energy fluctuation graphs for all models on each graph red line represents ΔG fluctuation for N-wild.

To determine the protein structure stability, ΔG values, as well as RMSD and ΔG fluctuation graphs, were considered and presented in Figs. 6, 7 and Supplementary Table S1. The average number of atoms in each of the systems, together with water and ions (Na^+^ and Cl^−^), is about 123,000 atoms, and the protein part of the systems is about 12,800 atoms. From the graphs of RMSD fluctuations (Fig. 6) for native N and all mutated models, it can be seen that the structure of the native N protein remained stable, while the mutant forms also showed instability of the structure, or stability at the beginning of the dynamics, then instability, and vice versa. Instability was observed especially in D3L, S186F, S197L, R203E, K256N, F307V models.

Using the trajectories obtained from molecular dynamics, we also calculated the binding free energy of the monomers that make up the dimer in the systems by the MM/PBSA and MM/GBSA methods. ΔG values results are presented in Supplementary Table S1. In Fig. 7, we present a series of graphs that depict the ΔG values computed using the MM/PBSA methodology across all systems under investigation. For these calculations, every 10th frame was selected, resulting in a total of 500 frames being considered for both the native and all associated mutated models.

From the data obtained by the MM/GBSA method (Supplementary Table S1), the strongest interaction of monomers in the dimer is observed in the case of the P326L mutation (ΔG = − 848.7848 kcal/mol), in the case when the following value is N wild-type (ΔG = − 765.8 ), the worst value is S202N (ΔG = 1331.8977), and strongest interaction by MM/PBSA data is Q418H (ΔG = − 643.6706), the weakest match with MM/GBSA data and it’s S202N (ΔG = 1477.9502). The more negative the value of ΔG, the more stable the system is, and vice versa. By data in Supplementary Table S1, according to MM/PBSA N wild-type ΔG = − 587.1434, and for Q418H ΔG = − 643.6706, T205A:ΔG = − 625.9514, P168Q:ΔG = − 615.9578, Q384H:ΔG = − 600.1673, P326L:ΔG = − 598.1866, D3L:ΔG = − 587.1434, which means that these mutations led to a significant stabilization of the dimer structure. And the mutations R203K/G204R (ΔG = 74.8293), S197L (ΔG = 490.5904), D63G (ΔG = 665.7818), K256N (ΔG = 751.8934), D377Y (ΔG = 994.0435), S202N (ΔG = 1477.9502) caused destabilization.

**Figure 8:**
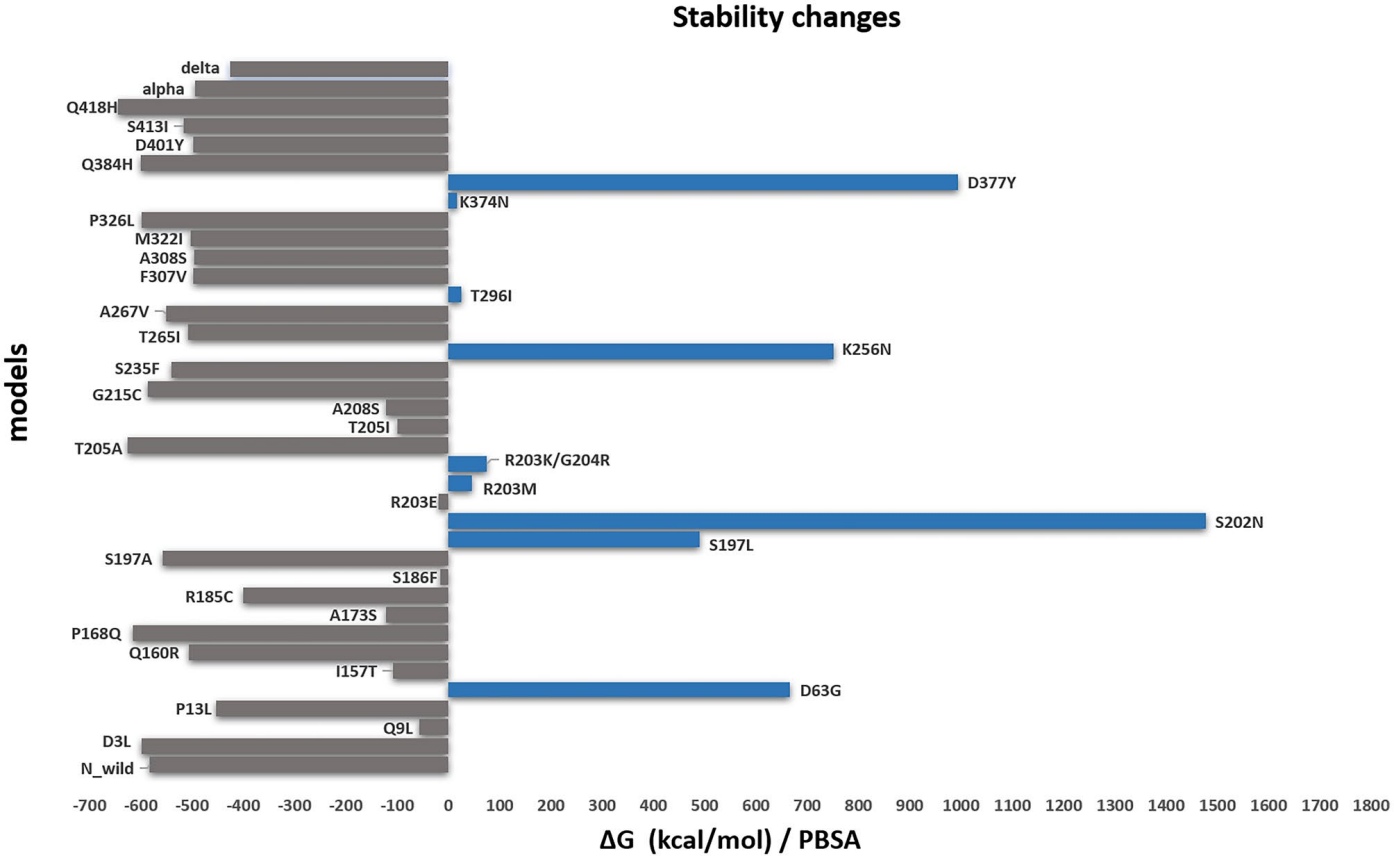
N protein structure stability changes calculated by the MM/PBSA method. The grey color bar represents stabilization, while the blue color bars represent destabilization.

As demonstrated by MM/PBSA analyses, it was found that the dimer structure was stable in case of 28 mutations (Q418H, T205A, P168Q, Q384H, P326L, D3L, G215C, S197A, A267V, S235F, S413I, T265I, Q160R, M322I, F307V, D401Y, A308S, alpha, P13L, delta, R185C, A173S, A208S, I157T, T205I, Q9L, R203E, S186F), and unstable in case of 9 mutations (K374N, T296I, R203M, R203K_G204R, S197L, D63G, K256N, D377Y, S202N), Fig. 8.

## Discussion

The N protein, widely distributed within the viral genome, assumes a key role in a spectrum of fundamental biological functions, including RNA packaging, viral assembly, replication, and transcription^1,2,4,6^. The multifaceted involvement of the N protein in these essential processes underscores its significance at the core of the virus life cycle. However, studying the structure of N protein is complicated by the presence of disordered regions characterized by inherent conformational flexibility and a lack of well-defined tertiary structures. The study of such disordered regions is of paramount importance, given their propensity to contribute to the functional versatility of the protein, particularly in mediating various protein–protein interactions and adaptations during viral processes.

The primary objective of our research was to elucidate the impact of mutations in the N protein on the stability of its dimeric conformation. The N protein attains its functional activity in a dimeric arrangement. Given the pivotal role of the N protein in interactions with both viral and host cell proteins, comprehending the potential influence of mutations on its dimeric structure assumes critical importance. Before scrutinizing the effects of the mutations, a prerequisite was the modeling of the complete structure of the N protein, a task rendered challenging by the presence of disordered regions that are inherently flexible. Employing the Alphafold2 technique, we executed the structural modeling and validated it by comparative analysis with the extant two X-ray structures of 2 domains: NTD and CTD, as well as cross-verification with models derived from cryo-EM.

The modeling encompassed both the monomeric and dimeric forms of the N protein, aiming to discern potential disparities between the two. Visualization of the tertiary structure and identification of secondary structural elements were pivotal in this comparative analysis. As illustrated in Fig. 1, we observed a pattern in the monomers that constitute the dimeric structure. Specifically, secondary structure elements were found to manifest in disorder regions within the monomers forming the dimer compared to the monomer model. An intriguing observation emerged when examining the dimeric structure itself—the disorder regions within the dimer exhibited a greater degree of assembly. We hold the view that the disorder regions in the protein tend to be more organized and assembled when part of a dimeric structure^7^.

Subsequent experimental investigations focused exclusively on the dimeric structure. The impact of a total 34 individual mutations, along with an additional 3 combined mutations, was systematically studied utilizing computational methodologies. This analytical approach afforded a comprehensive understanding of the nuanced effects of these mutations on the stability and conformational dynamics of the N protein's dimeric structure. The R203M/R203K, D377Y, D63G, G215C, G204R, D3L, S235F, and Q9L mutations included in our study are among the top 10 mutations of the N protein with the highest frequency worldwide^10^.

MD simulations and free binding energy calculations within the dimers showed that 9 of the 37 mutant models selected led to the destabilization of the dimer structure and 28 to stabilization. The R203K/G204R mutation caused destabilization, which was also shown by other authors^13,14,24^. These mutations have been particularly studied because it has been found in a larger number of lineages.

The escalated infectiousness of the R203K/G204R mutant virus was experimentally verified by Wu et al., as evidenced by heightened viral replication observed in the 203K/204R virus^13^. This enhanced replication was observed across various cell lines and primary upper airway tissues both humans and hamsters. The heightened efficiency in viral replication suggests a potential for increased virulence and fitness. Similar associations with heightened fitness and disease severity have been observed in other mutations linked to high infectivity, including D614G and N510Y in Spike (S) protein. Despite the distinct locations of the S protein and N protein, both being structural components of the virion, notable similarities between D614G and R203K/G204R exist concerning the consequences of the virus's properties.

In article from Mourier et al. has shown that R203K/G204R has higher viral loads in COVID-19 patients in Saudi Arabia^24^. R203K/G204R mutations increased along with the mutation of the Spike Y501N protein. R203K/G204R mutation increased oligomerization potential and RNA-binding affinity compared with wild-type N. Also, they showed that R203K/G204R mutations lead to significant changes in protein structure (destabilize) and potentially enhance the protein's ability to bind RNA and alter its response to serine phosphorylation events. In any case, the authors did not find an association between the R203K/G204R mutations' high load and mortality, because even though the virus had a high load, it did not affect mortality, since it is possible that other factors played a role, such as a change in the course of treatment, or a person's susceptibility to infection. the complications that occurred^24^.

Anyway, making definitive conclusions about the correlation between disease severity and mutation based on a single mutation within one protein is challenging. The reason is that strains typically possess multiple complex mutations in various proteins, both structural and non-structural. During our study, 63.8% of selected mutations were located in disorder regions, of which 60.86% were in the LKR part, which is involved in protein–protein interactions. About this, Abavisani et al. also note that the majority of N protein mutations occur in 164 to 205 AA and the second highest mutations frequency in N 205 to 246 AA, which also include in the basis of the disorder sections^10^.

The study by Oulas et al. demonstrates that the P13L mutation can influence both transmissibility and death rates. Specifically, this mutation is associated with a decrease in the number of deaths and cases per million^15,25^. For the P13L mutation, our experiments showed that it leads to structural stabilization.

Making definitive conclusions about the correlation between disease severity and mutation based on a single mutation within one protein is challenging. The reason is that strains typically possess multiple complex mutations in various proteins, both structural and non-structural, and more research is needed, because the same R203K/G204R mutation, which has been reported in several articles to affect virulence, pathogenesis, and fitness^10,11,13,24^, is found in the base together with the spike protein N510Y mutation, which also enhances viral attachment and infectivity^10,24^․

In conclusion, our findings hold significance in elucidating the tertiary structure of the N protein dimer and comprehending the consequences of mutations on its conformation. Our study provides insights into the behavioral dynamics of these mutations within the host cell. It is known from the literature that intrinsically disordered proteins (IDPs) undergo a disorder-to-order transition state during protein–protein interactions^7,26,27^. We advance a hypothesis suggesting that in the dimeric state characterized by protein–protein interaction, secondary structure elements appear in the disordered parts of the protein, and the protein is assembled in its tertiary structure. As illustrated in Fig. 1d and Supplementary Fig. S1, our findings indicate that, compared with N monomer secondary structure elements, in dimers, the secondary structure elements appear in disorder regions. And compared to the wild-type dimer N structure, models featuring the D63G, Q160R, S197L, S186F, S202N, S203E, R203M, K256N, T296I, S413I, Q418H, alpha and delta mutations (35,1% of all models) exhibit a reduced presence of secondary structure elements within the disordered regions (Supplementary Fig. S1). From which D63G, S197L, S202N, R203M, K256N, and T296I mutations (46,15%) led to the destabilization of the structure. This allows us to assume mutations that caused destabilization, are less likely to generate secondary structure elements in disordered regions of the protein.

Our results contribute not only to an understanding of the SARS-CoV-2 N protein but also offer valuable hints for the investigation of conservative proteins in other viruses within the same genus. Our research establishes a basis for proactive strategies designed to address the pathogenesis of this viral illness as the presence of disordered regions in viral proteins is commonly associated with viral infectivity and pathogenicity, as these regions typically play an essential role in the binding process with targets^7^.

Certainly, in the context of single protein mutations, drawing unequivocal conclusions regarding their impact on virus flexibility, virulence, and disease progression among patients is challenging. Comprehensive investigations are essential, extending beyond the examination of individual mutations within a single protein. Instead, a more intricate approach involves conducting studies at the level of multiple mutations within proteins that play crucial roles in the virus's life cycle. This encompasses an exploration of the collective effects of mutations across various proteins involved in the virus life cycle.

## Methods

### Modeling

To carry out the research, we used Alphafold2 version 2.1.2 for protein structure prediction^19,28^. The amino acid sequence of the SARS-CoV-2 N protein (UniProt ID: P0DTC9) was used for molecular modeling.

### Phosphorylation and mutations

All mutations were obtained using the ICM-Pro software^29^, and then the models were phosphorylated at the following sites—S23, S180, S186, S188, S194, S197, S201, S202, S206, T24, T198, T205, T265, T391^30^.

### MD simulations, analysis (MM/PB(GB)SA, RMSD)

The resulting systems were subjected to 100 ns molecular dynamics (MD) simulations using AMBER20^31,32^. All molecular dynamics (MD) simulations were conducted in water environments using the ff19SB force field for the solute, as it has been demonstrated to synergize optimally with the OPC water model, which offers enhanced accuracy^33–35^. The solvation box, housing the water molecules, was defined as a cubic periodic boundary to ensure homogeneous spatial distribution and to mitigate potential edge effects throughout the simulation trajectories. The integration step for the leap-frog algorithm was fixed at 2 fs. Simultaneously, the system was maintained at 309.75 K under standard atmospheric pressure.

The binding free energy was calculated by the MM/PB(GB)SA method, which is based on the determination of the energy of the protein–ligand complex, the individual energies of the protein and the ligand, and then the determination of the energy difference^36,37^.

Molecular dynamics simulations were executed across all the prepared systems, each spanning a duration of 100 ns. For every individual system, a consistent sampling rate was maintained, resulting in the acquisition of 5,000 frames. The binding free energy (ΔG) is calculated according to Eq. (1)^36,37^.

1 $$\Delta G={\Delta G}_{complex}-{\Delta G}_{receptor}-{\Delta G}_{ligand}$$

Since each system consists of an N protein dimer, the role of the receptor and the ligand were each of the monomers: receptor (monomer1)—the monomer with amino acid residues 1–419, and ligand (monomer2)—the monomer with amino acid residues 420–838, Eq. (2).

2 $$\Delta G={\Delta G}_{complex}-{\Delta G}_{monomer1}-{\Delta G}_{monomer2}$$

Typically, the quality of a model is assessed based on its energy or similarity to a reference structure—RMSD (usually atoms in the protein backbone). RSMD analysis of all obtained trajectories was performed using the CPPTRAJ program. RMSD can be used to identify significant changes in protein structure relative to the starting point. The equilibrium nature of the RMSD curve indicates that the protein was equilibrated^38^.

For studying structural rearrangements (loop-α-helix, loop-β-sheet, β-sheet-loop, and α-helix-loop) the PDBSum website was used^23^.

For visualization, we have used ICM-Pro software, and for graph building Gnuplot and ChimeraX have been used^39,40^.

### Supplementary Information

Supplementary Information.

## Data Availability

The datasets used and/or analysed during the current study available from the corresponding author on reasonable request.
