# Supplementary material for: Impact of mutations on the stability of SARS-CoV-2 nucleocapsid protein structure
Source: Sci Rep. 2024 Mar 11;14:5870. doi: 10.1038/s41598-024-55157-8 (PMC10928099; doi:10.1038/s41598-024-55157-8)
Supplement: Supplementary file 1 — Supplementary Information. [file 41598_2024_55157_MOESM1_ESM.pdf]

Impact of Mutations on The Stability of SARS-CoV-2 Nucleocapsid Protein Structure

Nelli Muradyan, Vahram Arakelov, Arsen Sargsyan, Adrine Paronyan, Grigor Arakelov*, Karen Nazaryan

Supplementary information

Model	RMSD (Å)	ΔG (kcal/mol) /GBSA	ΔG (kcal/mol) / PBSA	Impact on dimeric structure
**N_wild**	0	-765.8	-582.4621	-
**D3L**	2.703732	-643.0159	-597.5844	Stabilization
**Q9L**	3.492112	-23.8875	-56.738	Stabilization
**P13L**	17.41329	-468.4707	-453.3471	Stabilization
**D63G**	22.69168	425.8336	665.7818	Destabilization
**I157T**	11.08546	-189.2546	-107.953	Stabilization
**Q160R**	10.85181	-482.2883	-506.8819	Stabilization
**P168Q**	3.659702	-535.5657	-615.9578	Stabilization
**A173S**	7.445439	-99.2243	-121.7429	Stabilization
**R185C**	2.297269	-659.742	-400.7472	Stabilization
**S186F**	25.42511	-105.9902	-16.544	Stabilization
**S197A**	13.47089	-695.4409	-558.1588	Stabilization
**S197L**	22.92663	317.0991	490.5904	Destabilization
**S202N**	22.83753	1331.8977	1477.9502	Destabilization
**R203E**	23.73795	188.003	-19.8638	Stabilization
**R203M**	25.23095	-3.3787	45.5107	Destabilization
**R203K/G204R (gamma,omicron)**	8.804363	137.8287	74.8293	Destabilization
**T205A**	2.759875	-743.4905	-625.9514	Stabilization
**T205I (beta)**	12.76578	-347.7636	-100.1954	Stabilization
**A208S**	26.33809	-195.2891	-120.9124	Stabilization
**G215C**	13.92006	-606.787	-587.1434	Stabilization
**S235F**	20.94994	-595.6498	-539.8882	Stabilization
**K256N**	2.19617	282.1202	751.8934	Destabilization
**T265I**	10.76481	-527.9684	-507.0009	Stabilization
**A267V**	7.127691	-582.0163	-550.3871	Stabilization
**T296I**	13.79645	-7.7346	24.7335	Destabilization
**F307V**	16.16681	-493.572	-497.6513	Stabilization
**A308S**	14.489119	-513.1823	-496.6463	Stabilization
**M322I**	23.937444	-572.2682	-501.8638	Stabilization
**P326L**	12.55659	-846.7848	-598.1866	Stabilization
**K374N**	6.146784	-102.6273	17.2428	Destabilization
**D377Y**	29.12195	1084.4858	994.0435	Destabilization
**Q384H**	5.726878	-603.4073	-600.1673	Stabilization
**D401Y**	21.916738	-561.413	-497.0143	Stabilization
**S413I**	19.18349	-445.2662	-516.0638	Stabilization
**Q418H**	22.11971	-651.7179	-643.6706	Stabilization
**alpha**	18.747208	-499.2730	-493.5455	Stabilization
**delta**	30.765443	-388.4431	-425.2316	Stabilization

Table S1. RMSD scores for mutated models compared with native N protein, ΔG values for MMGB/PBSA

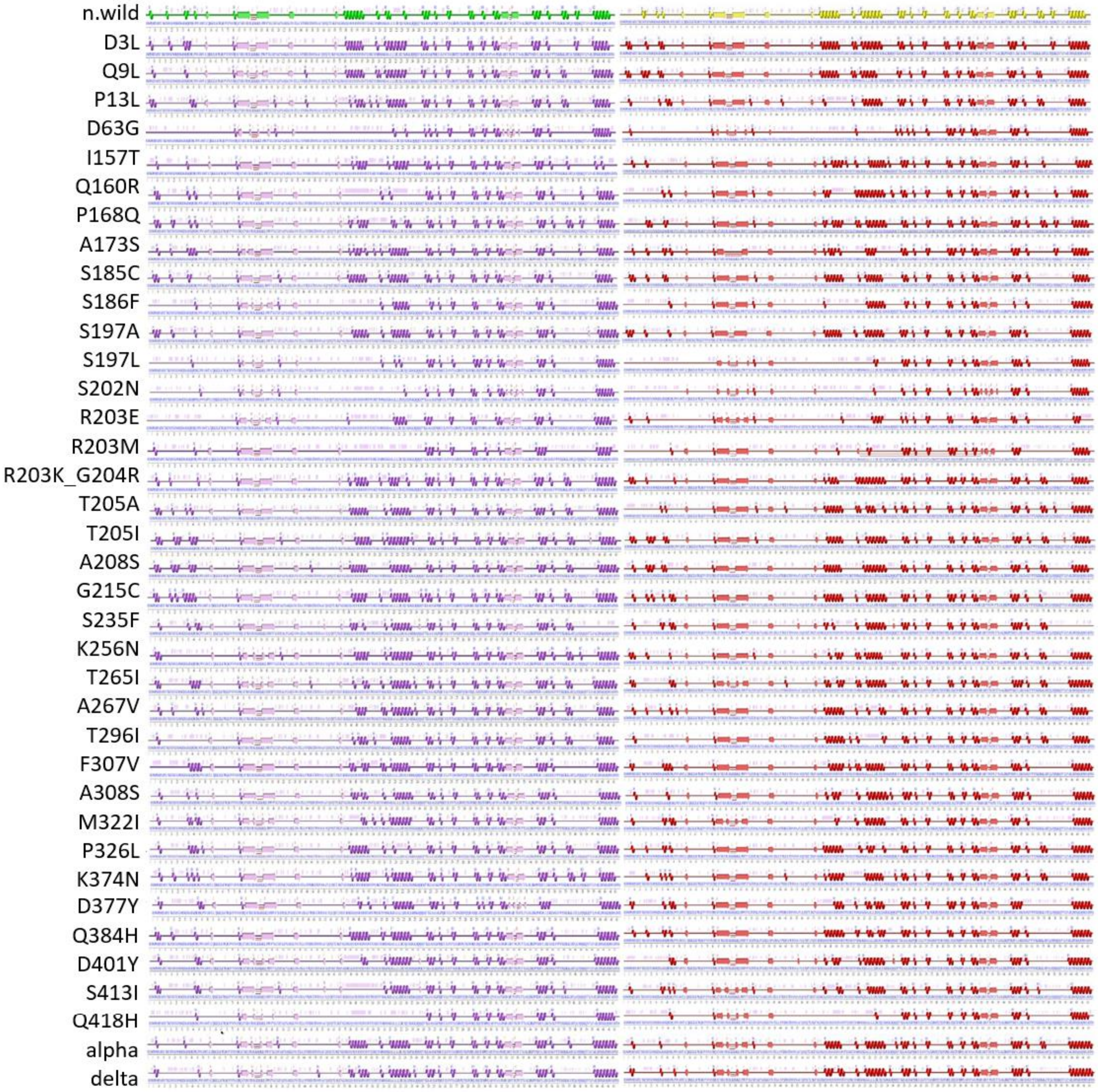

Figure S1. Structural rearrangements for mutant N proteins. for N_wild: in green - monomer 1, in yellow - monomer 2, for mutant forms: purple-monomer1, red-monomer2.
